# Reducing muscle weakness in nursing home residents: a study quantifying acceptance and feasibility of a formal training algorithm, and reliability of endpoint measures

**DOI:** 10.1007/s40520-025-03319-7

**Published:** 2026-01-15

**Authors:** Jonas Böcker, Ludwig Sachs, Michael Drey, Claudia Kaiser-Stolz, Wilhelm Bloch, Anja Dekant, Jörn Rittweger

**Affiliations:** 1 Institute of Aerospace Medicine, German Aerospace Center, Cologne, North-Rhine Westphalia Germany; 2 Specialist in Psychosomatic Medicine and Psychotherapy, Bad Endorf, Bavaria, Germany; 3 Department of Internal Medicine IV, LMU University Hospital, LMU Munich, Germany; 4 Department Molecular and Cellular Sports Medicine, German Sport University Cologne, Cologne, Germany; 5 Medical Director Cluster, Orpea Germany GmbH, Frankfurt am Main, Hessen Germany; 6 Department of Pediatrics and Adolescent Medicine, University of Cologne, Cologne, Germany

**Keywords:** Sarcopenia, Acceptance, Feasibility, Training algorithm, Training barriers

## Abstract

**Background:**

Sarcopenia is a growing problem, especially in nursing care. It is therefore mandatory to integrate measures such as resistance training to maintain muscle strength into nursing care.

**Aims:**

The aim of this study was to investigate the acceptance and feasibility of a novel training algorithm in a nursing home environment. Furthermore, the reliability of measurements for the diagnosis of sarcopenia was tested in the nursing home setting.

**Methods:**

Twenty-eight nursing home residents took part in the study, which encompassed two pre- and two post-examinations and a four-week training intervention. The training sessions were documented with regard to acceptance and feasibility as well as training motivation and intensity.

**Results:**

A combined acceptance and feasibility of at least 54% was shown, quantifying adherence of the residents to the training. The operational feasibility was 91% and the exercise performance feasibility of the residents was between 88% and 94.2%. All intraclass correlation coefficients showed at least a good reliability (all ≥ 0.84). Training motivation was higher when participants trained in a group (*p* = 0.007), but training intensity was greater when they trained individually (*p* < 0.001).

**Discussion:**

The main influencing factors for acceptance and feasibility were illness in general and a lack of motivation by the residents. Against the assumption, training was also possible during the weekends.

**Conclusions:**

In conclusion, the study shows that our proposed training algorithm is acceptable and feasible in a nursing home environment. In future, the efficacy of the training needs to be shown.

**Trial registration number:**

DRKS00030211; Date of registration: 2022-09-12.

**Supplementary Information:**

The online version contains supplementary material available at 10.1007/s40520-025-03319-7.

## Introduction

Sarcopenia is a rising problem world-wide, especially in light of changing demographics [1], which also has a major impact on nursing needs. The progressive loss of muscle strength is additionally aggravated by inactivity and lack of exercise, e.g. by being bedridden, due to reduced mechanical loading of muscle and bone [2, 3]. Decreased muscle strength leads to limitations in managing daily life independently [4], resulting in a loss of autonomy and reduced social participation. Altogether, this is significantly compromising quality of life at old age. Within this context, a factor that is crucially important in several ways is the ability to walk. Especially in nursing homes, physical inactivity and immobilization are widespread and pose great challenges for the nursing staff, for whom back pain prevalence of 41% [5] up to 69% [6] has been reported. Moreover, lack of activity has direct and indirect implications for health, well-being and autonomy of the residents [7]. Maintaining their independence for as long as possible, therefore, will relieve the staff on the one hand and improve the quality of life on the side of the residents.

**Fig. 1:**
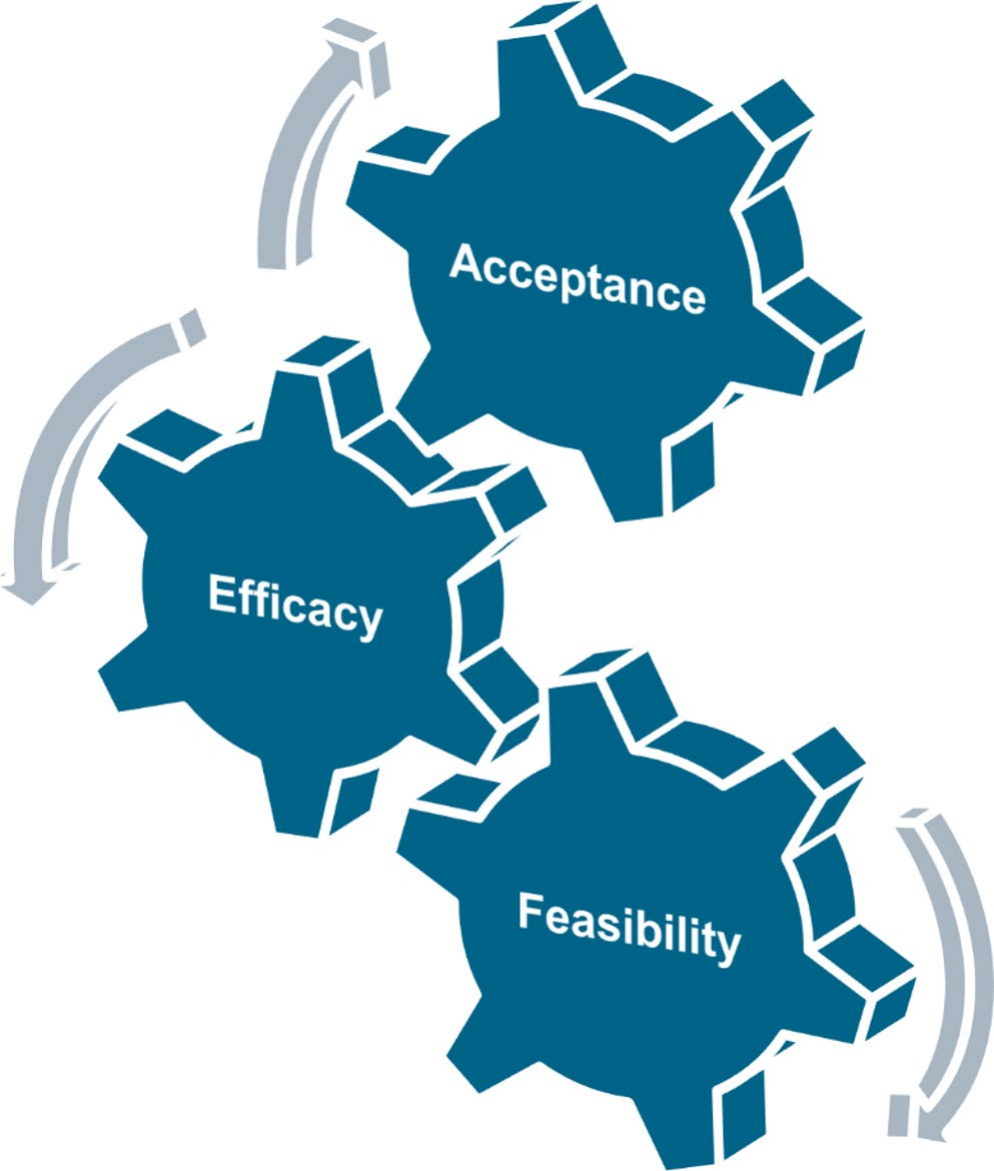
Schematic of the interdependency of acceptance, feasibility and efficacy. All three can be thought to work in series, so they jointly determine the effectiveness of a treatment or intervention. Not that in reality, these factors likely interact with each other, which further underlines the importance of feasibility and acceptance.

Resistance training is proposed to combat sarcopenia and improve mobility [8], and numerous training approaches have already been developed and tested in sarcopenic, elderly patients [7, 9–20]. Typically, resistance training relies on usage of free weights or machines, which was shown as feasible and accepted in the nursing home environment [21–24]. However, this training must always be accompanied by a qualified training or sport scientist, which raises further additional costs. But the aim should be to keep these costs and the effort for the nursing staff low in order to increase acceptance and feasibility while keeping a high standardization of training. It needs to be considered that nursing staff mostly are naïve to resistive training, which could influence the acceptance even more than the feasibility. In practice, however, feasibility, efficacy and acceptance are equally important, as they need to all work together for physical interventions to be efficient (Fig. 1).

These three factors jointly determine the effectiveness of a treatment or intervention clearly. Physically speaking, they work in series, which means that effectiveness can be modeled as the mathematical product. For example, if any of these factors is reduced to e.g. 50%, then the overall effectiveness is also lowered to 50%. In that respect, feasibility, efficacy and acceptance are all equally important. Despite this relationship, acceptance and feasibility are mostly neglected in the existing literature, which is confirmed by the small number of publications in this area [25]. Thus, it is mandatory to develop strategies, which are well accepted, feasible as well as efficient to counteract sarcopenic effects and to overcome the aforementioned barriers.

For this purpose, the competence network ‘immobilization-related muscle disorders’ (KNIMS; www.knims.de) has proposed a formal training algorithm that shall enable efficient physical therapy of sarcopenic patients [25]. As this is a novel approach, the aim of this study was the systematic quantification of the acceptance and feasibility of this formal training algorithm in the setting of a nursing home, focusing on the residents. It was the aim to reveal potential barriers reducing acceptance and feasibility, but generally it is hypothesized that such a training algorithm is accepted and feasible. Additionally, in order to arrive at sample size estimates for future effectiveness studies, we ventured to assess the reliability of the physical performance measures, as well as the responses of the nursing home residents to a 4-week training intervention.

## Materials and methods

Given that the primary aim was the assessment of acceptance and feasibility, the study was designed as a longitudinal non-controlled cohort study with a baseline assessment, a 4-week training intervention, and a final assessment (Supplementary Material Fig. 1). The study was conducted in five different nursing home institutions that are all operated by Orpea Deutschland (Orpea Deutschland, Frankfurt, Germany) and belong to the Orpea Group (Puteaux Cedex, Paris, France). The study had been approved by the Ethical committee of the medical chamber North-Rhine (approval number 2022047) as well as the Ethical committees of the medical chamber Hessen (approval number 2022-2953-zvBO) and Bavaria (approval number mb22024). It was registered at the German register of clinical trials (identifier DRKS00030211) before commencement, and it was carried out between October and December 2022.

### Participants

Inclusion criteria were residency in a nursing home, ability to stand freely, a score greater than 10 on the Mini Mental Status Test [26], and ability to perform the Timed-Up-And-Go-Test (TUG). The criterion of the Mini Mental Status Test was chosen, because guided interviews were conducted with the participants, which will be published in a separate publication and even participants with mild cognitive impairment could lead to incorrect conclusions in this case. Exclusion criteria were a TUG time < 20s, medically documented depression, and a history of one or more falls within the previous four weeks. We excluded participants with the ability to perform TUG in less than 20s as the training algorithm aims at groups of people who suffer from limited walking ability obtaining the acceptance and feasibility in this specific group. However, because the training algorithm is specifically aiming at maintaining or improving walking ability, the participants included under the above criteria reflect a wide range of physical limitations of nursing home residents. All assessments had been performed by the nursing staff prior to the inclusion examination by the medical staff of the study.

In total, 28 residents of five different institutions gave informed consent and participated in this study. Across the different institutions, the number of participants ranged from 2 to 10 residents per nursing home (Supplementary Material Fig. 1).

### Intervention

The nursing home residents trained according to the KNIMS algorithm and the goal was to carry out 5 training sessions per week from Monday to Friday over a period of four weeks. During each session, residents were asked to do three different exercises: First, squatting exercise, followed by lunges and single leg raises. Whilst the squatting exercises target leg extensor muscles and anti-gravity support, the lunges also aim at hip muscles and body trunk rotation, and the single leg raises target hip and trunk muscles with an additional challenge to balance. Each exercise can be adapted to the individual abilities of the residents through simple adjustments. This is possible through increasing the range of motion. Moreover, squatting and lunges can become harder by an ‘auxo-component’, which involves voluntary stiffening of muscle groups, either internally (ago-antagonist stiffening) or externally, e.g. by pretending to push both feet together. This component enables a safe way to increase the individual training intensity. Single leg raises, finally, can be made more challenging by additionally flexing the standing leg, up to a one-legged squat. The three exercises were supposed to be performed in the aforementioned sequence (squatting, lunge, single leg raise), in order to progress from easier to more difficult.

Participants were asked to aim for intensity of 16 (‘hard’ exercise) or above on the Rating of Perceived Exertion (RPE) scale [27] and was self-reported after performing the specific exercise. More detailed information regarding the KNIMS algorithm is described elsewhere [25]. All training sessions were instructed, guided and supported by briefed nursing staff (nursing staff, physiotherapists, social service). Staff was briefed on the KNIMS algorithm in a face-to-face meeting with at least two weeks’ notice. Any questions could be discussed with the study director either at the meeting or at any time thereafter. A handout with key information could be used to clarify any uncertainties during the intervention. Of note, training sessions could either be organized as individual training or group training, depending on preference and feasibility in each of the institutions. In some cases, the residents trained single training sessions in the group, but other training sessions were trained individually. Participants were therefore not generally divided into group or individual training.

### Assessing acceptance and feasibility

For this work, we have operationalized acceptance and feasibility as follows. Based on the fact that 5 training sessions per week were planned over 4 weeks, a total of 20 training sessions was set as the operational target. The operational feasibility (opF) was then defined as the number of training sessions offered by the staff, as assessed by documented training dates. The exercise performance feasibility (EPF) was assessed separately for each of the 3 exercises as the percentage of sessions in which the given exercise was performed. If an exercise could not be performed, it was assessed whether physical or mental barriers prohibited a performance. The combined feasibility and acceptance (cAF) was defined here as the percentage of the training sessions carried out by the individual participants in relation to the planned training sessions. The training sessions itself were documented by the instructing nursing staff including the date, the participant ID, the training performance (which of the three exercises could be performed and if no, whether physical or mental reasons prohibited an exercise including a more specific comment), the design of the session (individual or group training), the motivation (0: no motivation to 4: very high motivation) as well as the training intensity (RPE score) for each exercise.

In addition, acceptance was also assessed by qualitative methods via standardized interviews. However, these data will be published in a separate manuscript due to the excessive amount of information.

### Physical performance measures

We selected the three physical measures that are foreseen for making the diagnosis of sarcopenia (grip strength, body composition, gait speed) [28]. In addition, we chose to measure jumping mechanography, which has proven feasible and meaningful in geriatric settings [29, 30]. In 3 out of 5 institutions, we organized duplicate physical performance measurements at baseline and post-intervention, in order to assess the reproducibility of the measures. This was done with two testing sessions that were separated by 24 h at least.

Gait speed was measured over 4 m between two marks on the ground, giving 1 m for acceleration and 1 m for deceleration. The required time was measured by using a stopwatch, and gait speed was computed as 4[m] / t[s]. Participants were instructed to walk as if they would cross the street at a green traffic light, and they were allowed to use their habitual walking aids (e.g. zimmer frame, walking cane etc.).

A handheld dynamometer (Jamar Hand Dynamometer Hydraulic, Sammons Preston, Rolyon, Bolingbrook, IL, USA) was used to measure grip strength in kilograms [kg]. Participants were instructed to squeeze the dynamometer as forcefully as possible. Both sides were measured three times and all measured values were noted.

The body composition was assessed by an InBody S10 (InBody, Cerritos, California, US) in sitting or lying position due to personal comfort of the participant. The InBody S10 was developed especially for people with physical limitations. Out of the available variables, we focused on the percent body fat and the appendicular skeletal muscle mass (ASMM) as well as the appendicular skeletal muscle index (ASMI), which is used as parameter for diagnosis of sarcopenia [31].

To determine the relative power of the leg muscles [W/kg], jumping height [m] and the Esslingen Fitness Index [%] (EFI), which compares the results of the maximum power output during lift-off in relation to body weight with a healthy, age- and sex-matched reference group, a two-legged jump test was carried out on the participants using a Leonardo jumping platform (Novotec Medical GmbH, Pforzheim, Germany) with its manufacturer´s software for computing the output. The participants were instructed to squat down and immediately straighten again. And, if possible, to achieve a flight phase. If no flight phase was possible for a participant, the attempts that did not include a flight phase were also rated as valid. A total of 3 valid attempts were measured per participant (Supplementary Material Fig. 2).

### Data processing

The handwritten documentation of the training sessions as well as the handwritten results of gait speed and grip strength were digitized using LimeSurvey (version 3.22.2, LimeSurvey GmbH, Hamburg, Germany), which allowed plausibility checks during data entry. In addition, 10% of the digitized documentation was randomly checked for accuracy and completeness.

Furthermore, the results of the body composition and jump test were exported. Further processing of the data was then performed using R Studio (Posit Software, Boston, USA) and R (www.r-project.org) in its version 4.3.0 and the analysis was done as intention-to-treat method. In case of missing data, these data points were not interpolated.

For evaluation of jump test data, the individual averages over three trials were computed, because averages yield better reliability than selection of highest values [32]. For grip strength, however, we chose to select the maximum of three trials as it is the established procedure for sarcopenia diagnosis [28, 33].

In order to be able to make initial statements about effectiveness of the algorithm, the pre-post changes were analyzed with regard to the parameters defining sarcopenia [28].

For each measurement, values were aggregated as means of pre and post values, respectively, and effects were computed as percent changes (Δpc) of these aggregated values.

### Statistics

The reliability of the physical performance measures was computed by the coefficient of variation (CV) as well as the intraclass correlation coefficient (ICC) (R function “icc” of package “irr” in its version 0.84.1) based on a single-rater, absolute-agreement, One-way Random effects model [34, 35].

Learning effects between repeated baseline measurements (PRE1 vs. PRE2) were checked using a two-sided paired t-test using the t.test function of R. Prior to running this statistical test normal distribution and variance homogeneity were verified by using the shapiro.test and leveneTest R-function. If there was no normal distribution or variance homogeneity, the non-parametric Wilcoxon test was performed (R-function wilcox.test).

A linear mixed-effect model (LMM) was performed by using the lmer-function of the lme4-package (version 1.1–33) to test for factors that were associated with the physical performance parameters as well as any significant adaptation related to the training intervention. The analysis included the participants as random effects and the respective institution, which was also included in the model, as a cluster effect. Fixed effects of the model were the number of training sessions, the individual motivation averaged over all training sessions, time (PRE vs. POST), participant age, sex, body mass index (BMI) and RPE averaged over all training sessions and exercises.

In addition, a linear mixed model was carried out to determine the influencing factors of motivation and RPE. In the analysis included were the same random factors as in the previous analysis, but the fixed factors included the design of the training session (group training or individual training), age, sex and BMI. Additionally, the motivation was included in the analysis of influencing factors of RPE.

To compare the RPE values of the three exercises, a one-way Anova (aov-function) with TukeyHSD post hoc test (TukeyHSD function) was performed. The lm-function was used for assessing any time-depending linear relationship of RPE of the different exercises.

## Results

The mean age of the participants was 79.6 ± 13.9 years, mean weight 73.7 ± 15.0 kg, mean height 165.4 ± 8.2 cm and mean BMI 26.8 ± 4.5 kg/m² (15 females and 13 males). There were 8 drop-outs, and duplicated measurements were obtained in 17 participants at baseline and in 12 participants after the intervention (Supplementary Material Table 1).

In total, 6 participants showed sarcopenia (21.4%; 3 females, 3 males) based on grip strength and ASMI and one of these 6 participants (female) showed severe sarcopenia as the gait speed matched the sarcopenia criterion, too.

However, jumping performance indicated that 96% of all participants matched the sarcopenia criterion of reduced power. The pronounced deficit in vertical jump ability reflected by low readings for the EFI, which were 37.3% ± 19.7% for the females and 40.6% ± 26.3% for the males (Supplementary Material Table 2).

### Acceptance and feasibility

As per protocol, a total of 560 individual training sessions were to be offered (28 included participants * 5 training sessions per week * 4 weeks of intervention = 560 individual training sessions). However, Covid-19 infections and other illnesses on the side of staff and participants, as well as shortage of nursing staff led to restriction of this number. Thus, due to sick-leave, one of the five institutions reported to the study coordinator that they had to cut the number of offered training sessions down to two per week for three out of four weeks of the intervention phase (Supplementary Material Fig. 3).

That reduced the offered training sessions to 560–3 weeks of intervention * 3 training sessions per week * 6 participants in the specific institution = 506 effectively targetable sessions. In other cases, however, sick-leave, quarantine and lack of staff went unreported, so that we do not have a complete record of which training sessions were canceled because of staffing issues, and which sessions were missed due to participants’ unwillingness. On the other hand, it is also possible that some training sessions were performed but not documented. In 19 cases, by contrast, scheduled sessions that had been missed during the week were repeated during weekends.

As a result, a total of 275 training sessions of intended 506 training sessions have been performed and documented, yielding a documented cAF of 54%.

Of the 275 training sessions, 242 documentations had a date and could be assigned to an institution, thus could be included in the opF analysis. In two institutions training sessions were offered during the weekend resulting in more than the intended 20 training sessions (one offering 22 and the other 25 sessions). This resulted in an opF between 65 and 125%, with an average of 91% (SD 25.3%) across all institutions.

The EPF showed that squats (exercise 1) were performed in 259 (94.2%) of all 275 documented training sessions. Physical limitations as knee pain, weakness and falling were the reason in 8 cases (57.1%) and motivational limitations in 3 cases (21.4%) for missing squatting performance. Missing percentages occurred because of incomplete documentations. For the lunges, EPF was 92% (253 of 275 documented training sessions), although in 45.5% of the cases physical reasons (knee pain, dizziness) and 45.5% of the cases mental reasons (too strenuous, not confident, no motivation) prevented performance. For the single leg raise, the EPF was 88% (242 of 275 documented training sessions). This exercise could not be performed in 44.4% of the cases due to physical reasons (exhausted, too strenuous, knee pain, dizziness), 51.9% were mental reasons (no desire anymore, mentally not feasible). Table 3 of the Supplementary Material shows the cross-tabulation for further description of the EPF, where is shown that a participant, who could perform the lunge or the single leg raise, could do the squatting, which was the first exercise in all training sessions.

In general, cAF, opF and EPF were given across all institutions, but it needs to be mentioned that illnesses, especially of the nursing staff, as well as missing motivation of the residents were the key factors influencing the analyzed factors.

### Reliability of physical performance measures

Statistical testing revealed no learning effects between PRE1 and PRE2 for any of the physical performance measures (all *p* > = 0.12). The coefficient of variation (CV) ranged between 0.9% (ASMI) to 13.3% (Gait speed). According to Portney et al. (2009), ICC indicated good reliability for walking speed (0.84) and excellent reliability for all other measures (0.92 to 0.99) [36]. Observed effects ranged from − 5.8% for jumping performance to 1.7% for ASMM (Table 1).

**Table 1:** Overview of reliability and intervention effects

		Grip Strength		Jump	Gait Speed	Body Composition		
		Right [kg]	Left [kg]	rel Power [W/kg]	[m/s]	Percent Body Fat [%]	ASMM [kg]	ASMI [kg/m²]
CV [%]	PRE1/ PRE2	12.2	10.0	8.9	13.3	2.3	1.0	0.9
ICC	[95% Conf Int]	0.92 [0.84; 0.96]	0.96 [0.91; 0.98]	0.98 [0.95; 0.99]	0.84 [0.69; 0.92]	0.97 [0.93; 0.99]	0.99 [0.99; 0.99]	0.99 [0.99; 0.99]
Learning Effects	%change (p-values)	6.9 (0.12)	-0.5 (0.88)	2.9 (0.48)	0.9 (0.86)	1.5 (0.88)	0.05 (0.45)	0.7 (0.12)
Absolute values ± SD	PRE POST	19.5 ± 7.6 19.1 ± 8.0	19.4 ± 7.0 19.2 ± 6.9	10.5 ± 5.5 9.9 ± 5.3	0.74 ± 0.24 0.71 ± 0.23	35.4 ± 7.1 34.3 ± 8.2	16.8 ± 3.8 17.1 ± 3.9	6.2 ± 1.0 6.3 ± 1.0
Effect [%]	PRE/POST	-2.2	-1.2	-5.8	-4.7	-3.1	1.7	1.6

Coefficient of Variation (CV) and intraclass correlation coefficient (ICC) were calculated. The ICC is based on single-rater, absolute-agreement, One-way Random effects model. Learning effects are given as %differences between aggregated post and aggregated pre data

So, the reliability of the physical performance measures was given, whereas there was just a significant effect of the training intervention on percent body fat.

### Factors influencing training motivation and RPE

Analysis of participant motivation was available from 222 training documentations (Table 2), as some of the documentations showed missing dates (5 documentations), missing participant ID (6 documentations), the motivation was not specified (22 documentations) or training sessions were carried out at the weekend and therefore could not be assigned to a planned study day (20 documentations). The results of the performed linear mixed model for motivation shows an influence (*p* = 0.007) of the training organization, suggesting that motivation was higher for group training than for individual training (Table 2).

**Table 2:** Distribution of training motivation

	Overall (*N* = 222)	No Motivation (*N* = 5)	Low Motivation (*N* = 24)	Moderate Motivation (*N* = 70)	High Motivation (*N* = 94)	Very high Motivation (*N* = 29)	*p* values (LMM)
Sex							
Female	129 (58.1%)	3 (60.0%)	13 (54.2%)	32 (45.7%)	59 (62.8%)	22 (75.9%)	0.39
Male	93 (41.9%)	2 (40.0%)	11 (45.8%)	38 (54.3%)	35 (37.2%)	7 (24.1%)	
Age (years)							
Mean (SD)	78.9 (14.5)	85.4 (5.46)	78.9 (12.4)	85.8 (8.89)	77.5 (14.3)	65.9 (18.9)	0.35
BMI (kg/m²)							
Mean (SD)	26.4 (3.97)	24.4 (2.57)	29.2 (5.09)	26.2 (3.97)	26.0 (3.50)	26.4 (3.90)	0.10
Training Design							
Individual	121 (54.5%)	2 (40.0%)	17 (70.8%)	46 (65.7%)	44 (46.8%)	12 (41.4%)	0.007^a^
Group	73 (32.9%)	0 (0%)	5 (20.8%)	12 (17.1%)	40 (42.6%)	16 (55.2%)	

^a^Motivation significant higher while group exercising

The table includes the training motivation of all included participants during the entire training intervention, and its relation to anthropometrics and training. A linear mixed model for the motivation was performed, shown are the p-values for the fixed effects

According to the evaluation, RPE was influenced by the training design (*p* < 0.001; RPE higher for individual training) and BMI of the participant (*p* = 0.018; participants with higher BMI had a higher RPE) (Table 3).

**Table 3:** Results of the linear mixed model for the training intensity (RPE)

		RPE	
Random Effects (Variances)	Participant	1.00	
	Institution	1.62	
	Residual	1.63	
		Beta	p
Fixed Effects (p-values)	(Intercept)	7.49	0.043
	Training Design	-1.06^a^	< 0.001^a^
	Age	0.03	0.34
	Sex	-0.80	0.29
	BMI	0.21	0.018^b^
	Motivation	0.01	0.94

^a^RPE significant higher for participants exercising individually

^b^RPE significant higher for participants with higher BMI

As random effect the participant and residuals, as cluster effect the institution. For these parameters the variances are shown. The fixed effects (Beta- and p-values) are training design (individual training for group training), age of the participant, sex, body mass index (BMI) as well as motivation

### Factors associated with physical performance

Participant motivation was positively related to the body mass specific vertical jump power during the jump test (*p* = 0.027). Additionally, BMI was negatively related to power output (*p* = 0.018), but positively related to percent body fat (*p* = 0.005), ASMM (*p* = 0.002), and ASMI (*p* < 0.001), respectively. Furthermore, age was negatively related to percent body fat (*p* = 0.035), men had greater grip strength (right: *p* = 0.016; left: *p* = 0.001) and significantly greater ASMM (*p* < 0.001) and ASMI (*p* < 0.001). Finally, participants with a higher mean RPE had greater percent body fat (*p* = 0.044, Supplementary Material Table 4).

### Effects of training

**Fig. 2:**
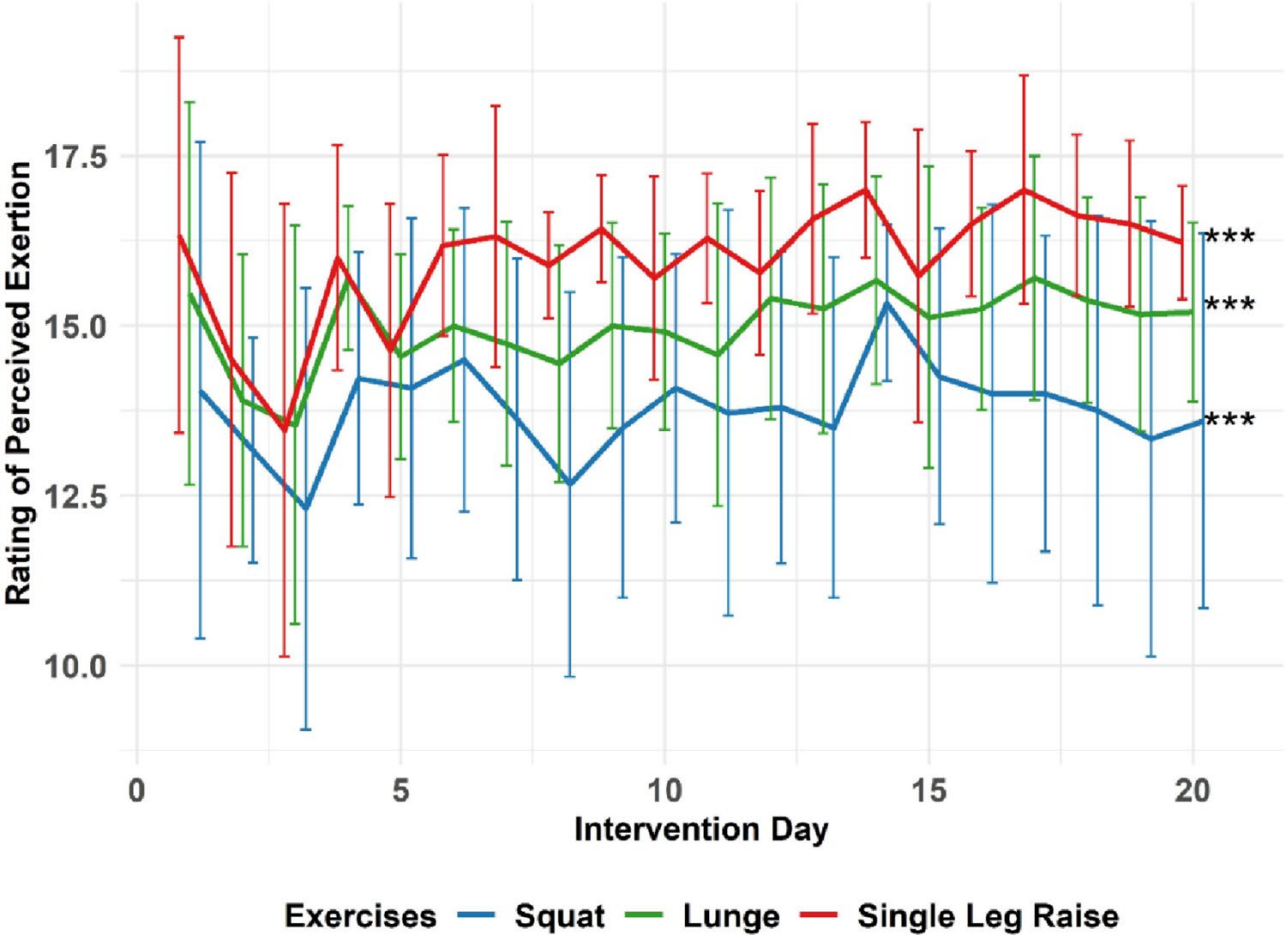
Progress of Rating of Perceived Exertion (RPE) during the 4-week training intervention. The figure shows the progress split by the different exercises. ***Anova and Tukey-Post-Hoc test showed that there were differences between squatting and lunges, squatting and single leg raise, and lunge and single leg raise, respectively (*p* < 0.001). Linear regression analysis showed correlation of RPE with time for Lunge (*p* = 0.03, *r* = 0.45) and for Single Leg Raise (*p* = 0.006, *r* = 0.56), but not for Squat (*p* = 0.44). The drop during the third study day was not explainable by the available data

Linear mixed models indicated a reduction (*p* = 0.046) of body fat (1.09% absolute and 3.79 ± 6.00% relative) from PRE to POST, but not for any other measure (Supplementary Material Table 4). The RPE showed differences (*p* < 0.001) between the three performed exercises, with mean values of 13.8 ± 2.5 (squat), 14.9 ± 2.0 (lunge) and 15.9 ± 2.1 (single leg raise) (Fig. 2).

Results of the linear regression model showed time trend for lunge (*p* = 0.03) and single leg raise (*p* = 0.006) with *r* = 0.45 (lunge) and *r* = 0.56 (single leg raise), but no time trend for squats (*p* = 0.44).

## Discussion

The study cohort represents an institutionalized clientele with general signs of age-related muscle loss. Six participants showed sarcopenia and one out of these 6 participants showed severe sarcopenia. In total, 46% of the participants have reduced grip strength, 48% of the participants have a reduced ASMI, and 62% of all participants had a reduced gait speed, respectively. Clearly, that group is physically compromised and requires treatment. This view is also supported by EFI-values that were as low as 37.7 ± 19.7% and 40.6 ± 26.3%, which is less than half the neuromuscular power that would be typically found in physically competent people of the same age and sex [37]. It is widely accepted and shown that targeted strength training can counteract loss of muscle strength, regardless of age [11] and if the algorithm improves one or more parameters used to diagnose sarcopenia, it can be assumed that it represents a useful therapeutic approach to combat sarcopenia. Therefore, in the authors’ opinion, it is important that a training program is developed that is both accepted as well as feasible in a nursing home environment.

### Acceptance and feasibility

Whilst the main focus of this work is on acceptance and feasibility, we further differentiated between (1) combined acceptance and feasibility (cAF), (2) operational feasibility (opF) and (3) exercise performance feasibility (EPF). Of note, the study was being conducted between October to December 2022, when a huge Covid-19 wave struck Germany. Accordingly, the main factors that interfered with cAF were acute illness of staff or participants (mostly Covid-19, plus some other reasons) and Covid-19-quarantine. Therefore, the cAF-figure of 54% obtained in this study is probably underestimating the acceptance that would be possible outside a pandemic. In the literature, adherence is usually used as a corresponding synonym in the context of acceptance. Thus, different studies report adherence of 66–90% [23], > 70% [12], 83% [38], and 84% [13] to training programs, respectively, depending on the duration of the training intervention, the participants, the organization of the training, and the training design. As stated before, our finding of 54% cAF would probably higher outside a pandemic and therefore would be consistent with previous findings. In our study, 57.1% (16 participants in total) performed more than 50% of the intended 20 training sessions and 35.7% (10 participants in total) even performed more than 75% of the planned sessions. Factors that resulted in high cAF were positive alternation through new exercises, social contacts, individual support, positive effects of the training and qualification of the instructor [23, 39]. The exercises of the training algorithm were not varied during our training intervention, so this factor played no role for cAF, although we achieved comparable acceptance.

For the reasons explained in the introduction, therapeutic resistance is needed most when and after people are sick and bed-ridden. Taking these findings and considerations together, it seems mandatory to offer at least 5 therapeutic strength training sessions per week so that nursing home residents can realistically perform a minimum of 3 weekly sessions.

Another important question is how sustainable acceptance and feasibility would be in the long run. In the present study, three participants dropped out of the study due to a lack of motivation (Supplementary Material Fig. 1), and the overall drop-out rate was 35% (10 out of 28), which compares well to existing literature [21]. To achieve the long-term goals of the intervention, it is therefore essential to minimize the dropout rate and maintain the residents’ motivation. Involving relatives to leverage their positive influence would be one way to achieve this [40]. Furthermore, motivating examples, such as residents who demonstrated a significant improvement in performance after training, would be a valuable addition. Additionally, if possible, training should be offered in a group setting, as social participation also has a positive impact on motivation. It was already shown that a drop-out rate of 20% was possible for an intervention of six months where training was self-directed after an introduction without supervision [10].

In addition to cAF, the opF of such a training algorithm, especially in a nursing home setting, also plays a crucial role [25]. In our case, we quantified the opF by putting the offered training sessions in relation to the planned training sessions. If we include all training sessions, we find an opF of 91%, whereas there are large differences between the five institutions. The main reason for this difference between the institutions is the availability of staff. For example, in one institution there was a high level of sick leave, mainly due to a Covid-19 outbreak, including the person responsible for conducting the study at the institution. This resulted in bottlenecks in the staff who were supposed to take over the guidance of the training, as well as a lack of organization of the further course of the intervention. Contrary to our original planning, training could also be offered and carried out on weekends. This resulted in an opF of > 100% in some institutions. We therefore conclude that the KNIMS-algorithm is operationally very feasible in the setting of a nursing home. To maintain this opF over a longer period of time, strategies to overcome staff turnover and seasonal illnesses need to be considered. The goal should be to integrate the training into everyday routines, for example, by expanding existing activities with the training algorithm. One advantage of the algorithm is that it can also be led by unqualified personnel, which facilitates coverage in case of illness or staff turnover. Furthermore, it is important to create the possibility for new employees to be trained by experienced staff, thus avoiding the need to rely on external personnel. Another factor to improve feasibility is a precise definition of responsibilities. It is important to clarify who is responsible for organizing implementation within the facility and in the event of illness or absence, this role should be able to be taken on by a deputy.

For the EPF, it was documented that the squatting could be performed by 94.2% of the documented cases, the lunge by 92% and the single leg raise by 88%. One reason for the reduced EPF for single-leg raise was exhaustion, which is precisely the goal of the algorithm to provide an adequate training stimulus. But the main factor limiting EPF were primarily mental factors, which could be overcome by a higher intrinsic motivation.

In general, in the course of the evaluation, we found that quantifying acceptance and feasibility is very challenging and complex. This analysis offers a first approach to quantify these parameters, but a guideline for the standardized quantification of acceptance and feasibility in the implementation of a training intervention for frailty persons would be useful. From our point of view main factors, which need to be considered in a standardized quantification would be: Detailed information about the training program (e.g. target group, exercise description), factors influencing acceptance (e.g. subject group, inclusion/exclusion criteria, season of the year, duration of training intervention), feasibility (e.g. planned number of training sessions, documentation of training sessions, number of staff members), EPF/applicability (e.g. number of assessing intensity/exhaustion, reasons for prematurely quitting).

### Reliability, effects and sample size estimations

The secondary objective of this study was to provide a basis for planning of future effectiveness studies. To this purpose, we have examined the reliability of physical performance measures suitable for diagnosis of sarcopenia [28], as well as the intervention-related effects.

None of the selected measures depicted a learning effect and their CV was generally below 10%, except for gait speed (13.3%). Reasons for the somewhat poorer performance of gait speed may include using a manual stopwatch and the fact that participants were allowed to use their habitual walking aids. The CVs of grip strength and jumping performance are also higher than those of the body composition measures (CV ≤ 2.3%). Clearly, functional tests will always be subject to behavioral aspects such as daily mood (Table 1). Of note, CV around 3% or lower for body composition measures is also confirmed by the literature [41].

However, ICC for gait speed is still suggesting good reliability (0.84) and we found excellent reliability for all other measures (ICC > 0.92) (Table 1) [36], which is also confirmed in the existing literature [29, 42, 43].

However, the study failed to provide meaningful effect sizes for the intervention outcome. The reasons for this include the lack of control group, the relatively high heterogeneity between participants, and the short intervention period of four weeks. Whatever the reasons, it seems that pre-post data acquired in his study do not allow a trustworthy sample size estimation for future effectiveness studies. However, existing longitudinal studies report effect sizes of 0.29 for grip strength [44], 0.2 for ASM [45], 0.29 for gait speed [44], and 3.82 for jumping performance [46]. Using this information we performed sample size estimations with the pwrss.t.2means function of the pwrss-package (version 0.3.1) with a significance level of 0.05 and power of 80% for non-paired samples. We arrive *N* = 378 for studies with grip strength as primary outcome [44], an estimate of *N* = 788 for ASM [45], of *N* = 366 for gait speed [44], and of *N* = 6 for jump [46] for future studies. It should be noted that the sample size estimation for the jump test is a best-case scenario, as the referred literature is based on master athletes who participate in regular training.

### Exercise exertion and motivation

The planned training intensity of 16 RPE was not reached in this study. The value averaged 14.8 across all exercises and even the RPE for the single leg raise averaged 15.9, which was below the intended value. Accordingly, in future studies about effectiveness of the algorithm, it is important to increase the intensity by using the possibilities offered by the KNIMS algorithm, although it has already been shown that improvements can also be achieved with intensities between 12 and 14 [47].

Another important finding of the analysis is that the training intensity was greater during the individual training, but the motivation of the participants was significantly better during group training. Since motivation has a great influence on acceptance as well as feasibility, the focus should therefore be placed on group training in the future. Only those residents who personally exclude group training or need intensive support should be trained individually.

### Training efficacy and physical performance

A secondary objective of this study was to obtain an initial assessment of the efficacy of the training algorithm. The effects that were observed after four weeks of intervention were to be used as a basis for sample size estimation. On this basis, a future study can be planned that focuses on the effectiveness of the algorithm. Nevertheless, it should be noted that even a short intervention of 4 weeks was associated with a significant decrease in percent body fat. Other studies have also shown effects after such a short intervention [48]. Nevertheless, a significantly longer duration should be chosen for a future study with regard to effectiveness.

### Limitations

Within this study there were two main limitations: firstly, the Covid-19 pandemic heavily impacted study conductance, and secondly, a lack of reporting of non-performed (and potentially also performed) training sessions. Both of these limitations lead to an under-estimation of cAF. However, even if we use the obtained figure of 54% and apply it to 5 training sessions per week, we arrive at 2 to 3 effective sessions per week, which is generally sufficient for building up muscles. Countermeasures to prevent muscle loss need to aim to increase that number. The important lesson here for future studies is to take better care of documenting training session. In the present study, documentation and performance of training sessions was assigned to the same person, which turned out to be problematic when staff were sick or in quarantine. One way to improve documentation could consist in assigning performance and documentation to different persons. Also, it turned out in the course of this study that the data collection lead (JB) was not organizationally embedded into the nursing homes, which hindered time-efficient communication, direct access to residents and their data etc. Hence, a secondary recommendation for future studies would be to organizationally embed the data collection lead into the nursing home.

An additional limitation was the selection of participants. Only residents who met the inclusion criteria were selected and participation was voluntary. It is therefore quite possible that primarily those residents who were more motivated to exercise took part. However, this can be countered by the fact that the majority of dropouts (*N* = 5, Supplementary Material Fig. 1) stated a lack of motivation as the reason for leaving the study. It should also be mentioned that in group training under supervision, the motivation of the instructor has an influence on the motivation of the group. As the staff who led the training were able to voluntarily decide to participate, we assume that the motivation to lead the training was roughly similar. The results of the guided interviews, which will be published separately, will provide more precise information on this aspect. Group dynamics, which can have both a positive and a negative effect on acceptance, must also be taken into account. In this study, it was recorded whether training was carried out individually or as a group, but it was not documented whether the group always consisted of the same members or whether they changed. This needs to be recorded in future studies in order to be able to assess the aspect of group dynamics and the influence of individuals.

### Strengths

To our knowledge, this study is the first attempt to quantify the acceptance and feasibility of a training algorithm in a nursing home, taking many factors into account. In the course of the study, many factors influencing acceptance and feasibility became apparent and can be taken into account in the planning of future studies and the implementation of training interventions. The inclusion of five different nursing homes made it clear that there are differences between the institutions and that results from one nursing home cannot necessarily be transferred to other facilities.

## Conclusion

The study has shown that the KNIMS algorithm is generally accepted by the residents of a nursing home and feasible. The number of 54% is a substantial under-estimate (mostly due to the Covid-19 pandemic), and we expect that rates of 80% to 90% could be achievable. The vast majority of participants was physically able to perform the three KNIMS-exercises. As expected, the single leg raise was found to be more difficult than the lunge, which in turn was more challenging than squatting, and rating of perceived exertion was lower than the prescribed range. Taken together, this means that KNIMS-algorithm training allows for exercise progression within this setting. To do this, it is also necessary to consider the motivation of the residents. On the one hand, the study has shown that motivation is higher when training in a group, and on the other hand, extrinsic motivation by family and friends can lead to increased combined acceptance and feasibility. The physical performance measure used have high reliability, making them suitable for future studies of effectiveness. Overall, we conclude that application of the KNIMS-algorithm has proven feasible and acceptable for studying its effectiveness for therapeutic resistance training against sarcopenia and immobilization-related muscle disorders.

## Supplementary Information

Below is the link to the electronic supplementary material.

Supplementary Material 1

## Data Availability

The datasets used and/or analyzed during the current study are available from the corresponding author on reasonable request.
