# Supplementary material for: Reducing muscle weakness in nursing home residents: a study quantifying acceptance and feasibility of a formal training algorithm, and reliability of endpoint measures
Source: Aging Clin Exp Res. 2026 Jan 15;38(1):68. doi: 10.1007/s40520-025-03319-7 (PMC12886320; doi:10.1007/s40520-025-03319-7)
Supplement: Supplementary file 1 — Supplementary Material 1 [file 40520_2025_3319_MOESM1_ESM.docx]

# Supplementary Material

**Reducing muscle weakness in nursing home residents: A study quantifying Acceptance and Feasibility of a Formal Training Algorithm, and Reliability of Endpoint Measures**

Jonas Böcker^1^, Ludwig Sachs^1,2^, Michael Drey^3^, Claudia Kaiser-Stolz^1^, Wilhelm Bloch^4^, Anja Dekant^5^, Jörn Rittweger^1,6^

^1^Institute of Aerospace Medicine, German Aerospace Center, Cologne, North-Rhine Westphalia, Germany; ^2^Specialist in psychosomatic medicine and psychotherapy, Bad Endorf, Bavaria, Germany; ^3^Department of Internal Medicine IV, LMU University Hospital, LMU Munich, Germany; ^4^Department Molecular and Cellular Sports Medicine, German Sport University Cologne, Cologne, Germany; ^5^Medical Director Cluster, Orpea Germany GmbH, Frankfurt am Main, Hessen, Germany; ^6^Department of Pediatrics and Adolescent Medicine, University of Cologne, Cologne, Germany

Corresponding Author:

Jonas Böcker

Linder Höhe, 51147 Cologne, Germany

Email [jonas.boecker@dlr.de](mailto:jonas.boecker@dlr.de)


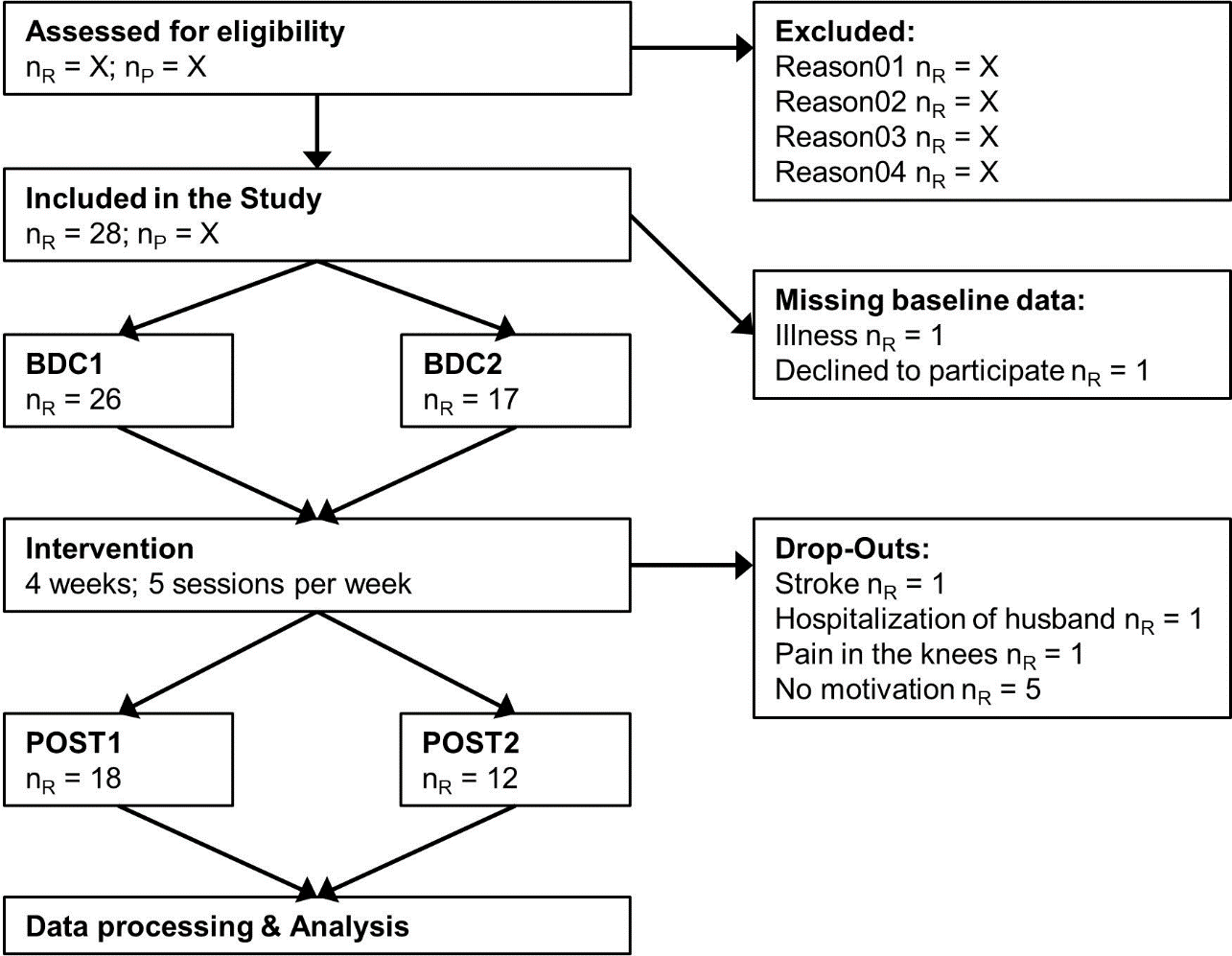


Figure 1: Consort diagram showing the study design

The diagram shows the included participants and the drop-outs during the training intervention as well as the study flow. n_R_: number of residents; n_P_: number of nursing staff; BDC: baseline data collection; POST: post-intervention data collection. X: no exact documentation/number available.


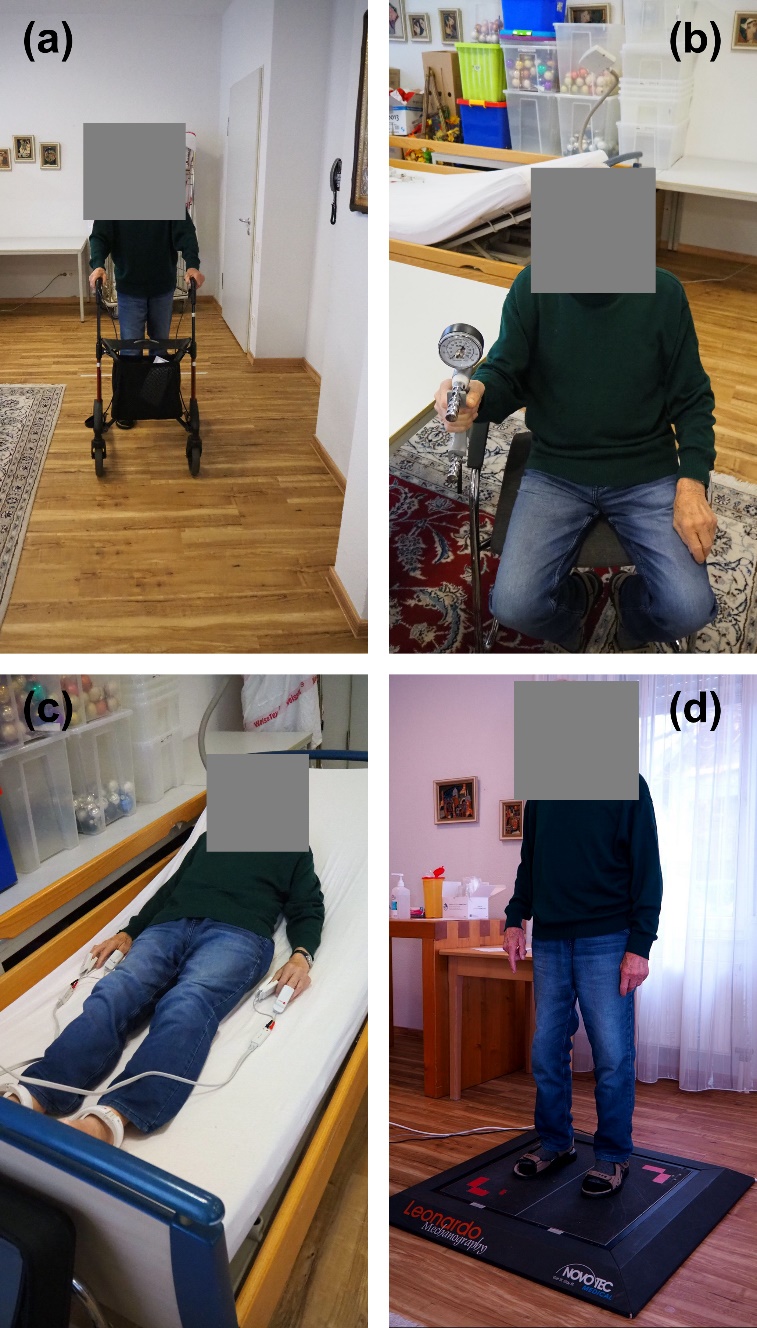


Figure 2: Pictures of the four measurements pre- and post-intervention

(a) 4m Gait speed test, (b) Grip Strength of right and left hand, (c) Analysis of body composition via bio-electric impedance analysis in lying position, (d) countermovement jump test.


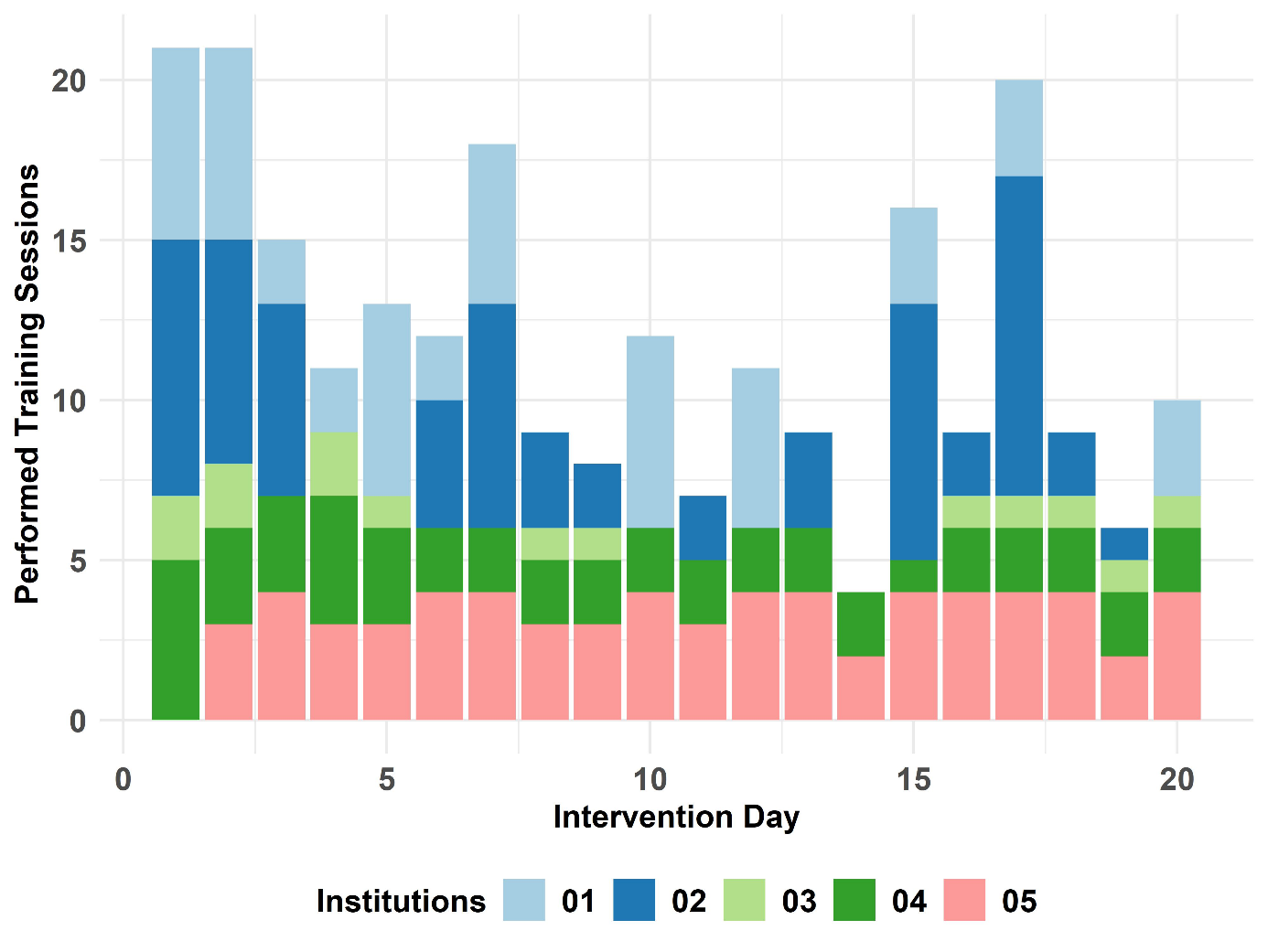


Figure 3: Number of performed training sessions separated by intervention days Monday to Friday and Institutions

Institution 01 was the institution with a Covid-19 break out, consequently offering just 2 training sessions per week from week 2 to 4. Trainings performed during the weekends were excluded as well as incomplete training documentations. In total, this figure includes 241 training documentations.

Table 1: Number of datasets included in the statistical analysis

| Parameter | | PRE1 | PRE2 | POST1 | POST2 |
| --- | --- | --- | --- | --- | --- |
| Grip Strength | Right | 26 | 17 | 17 | 11 |
|  | Left | 26 | 17 | 18 | 12 |
| Jump |  | 22 | 16 | 17 | 12 |
| Gait Speed |  | 26 | 17 | 18 | 12 |
| Body Composition |  | 25 | 15 | 16 | 11 |
|  |  |  |  |  |  |

Differences between PRE1 and PRE2 as well as between POST1 and POST2 because it was only in 3 out of 5 institutions possible to perform two measurements before and two measurements after the intervention. General differences between PRE and POST because of drop-outs, illnesses, lack of motivation, fear of failure, fear of the operator

Table 2: Baseline values of sarcopenic diagnostic criteria and jump

|  | Female | Sarcopenia Threshold | Male | Sarcopenia Threshold | %Matching Sarcopenia Criterion |
| --- | --- | --- | --- | --- | --- |
| Grip Strength [kg] | 15.5 ± 5.7 | <16.0 | 24.7 ± 8.7 | <27.0 | 46 |
| ASMM [kg] | 17.6 ± 3.7 | <15.0 | 23.0 ± 3.9 | <20.0 | 32 |
| ASMI [kg/m²] | 6.7 ± 1.1 | <5.5 | 7.9 ± 0.8 | <7.0 | 48 |
| Gait Speed [m/s] | 0.76 ± 0.29 | <0.8 | 0.73 ± 0.16 | <0.8 | 62 |
| Jump Power [W/kg] | 9.2 ± 6.5 | <19.0 | 9.6 ± 4.6 | <23.8 | 96 |
| Jump Height [cm] | 6 ± 5 | - | 6 ± 5 | - | - |
| EFI [%] | 37.3 ± 19.7 | - | 40.6 ± 26.3 | - | - |
|  |  |  |  |  |  |

Data are given as mean and standard deviation. %Females/Males below Threshold indicates how many of the participants relatively fulfill the indication for sarcopenia of this specific parameter. ASMM is the appendicular skeletal muscle mass (lean mass of arms and legs) and ASMI is the appendicular skeletal muscle index as assessed via bio-electrical impedance. EFI is the Esslingen Fitness Index, which is the maximum power output during the lift-off phase normalized for body mass, sex and age, with 100% being the predicted value.

Table 3: Cross-tabulation of the dependency of the single training exercises

|  | Squat | Lunge | Single Leg Raise |
| --- | --- | --- | --- |
| Squat | - | 251 (96.9%) | 241 (93.1%) |
| Lunge | 251 (99.2%) | - | 239 (94.5%) |
| Single Leg Raise | 241 (99.6%) | 239 (98.8%) | - |
|  |  |  |  |

This analysis is used for further description of the exercise performance feasibility. In the rows the independent variables, in the columns the dependent variables

Table 4: Factors influencing the physical performance measures

|  |  | Grip Strength | | | | | | | | Jump | | | | Gait Speed | | | Body Composition | | | | | | | | | | |
| --- | --- | --- | --- | --- | --- | --- | --- | --- | --- | --- | --- | --- | --- | --- | --- | --- | --- | --- | --- | --- | --- | --- | --- | --- | --- | --- | --- |
|  |  | Right [kg] | | | | Left [kg] | | | | | rel Power [W/kg] | | [m/s] | | | | | Percent Body Fat [%] | | | | ASMM [kg] | | | | ASMI [kg/m²] | |
| Random Effects | Participant | 10.47 | | | | 1.10 | | | | | 5.39 | | 0.01 | | | | | 2.37 | | | | <0.001 | | | | 0.02 | |
|  | Institution | 32.89 | | | | 32.82 | | | | | 9.92 | | 0.04 | | | | | 19.07 | | | | 5.20 | | | | 0.11 | |
|  | Residual | 4.97 | | | | 2.28 | | | | | 4.47 | | 0.01 | | | | | 2.76 | | | | 0.15 | | | | 0.02 | |
|  |  | Beta | | p | | Beta | | p | | | Beta | p | Beta | | p | | | Beta | | p | | Beta | | p | | Beta | p |
| Fixed Effects | (Intercept) | -1.22 | | 0.94 | | 51.35 | | 0.001 | | | 8.22 | 0.45 | 1.47 | | 0.019 | | | 12.65 | | 0.29 | | 10.11 | | 0.06 | | 2.18 | 0.03 |
|  | Sessions | -0.51 | | 0.07 | | 0.39 | | 0.11 | | | -0.24 | 0.16 | 0.001 | | 0.88 | | | -0.29 | | 0.15 | | 0.01 | | 0.13 | | -0.006 | 0.67 |
|  | Motivation | 1.13 | | 0.61 | | 0.82 | | 0.67 | | | 3.37 | 0.027 | 0.03 | | 0.69 | | | 1.41 | | 0.38 | | -0.38 | | 0.61 | | -0.01 | 0.91 |
|  | Day (Pre/Post) | -0.90 | | 0.22 | | -0.32 | | 0.52 | | | -0.51 | 0.46 | -0.03 | | 0.31 | | | -1.23 | | 0.046 | | 0.20 | | 0.14 | | 0.07 | 0.18 |
|  | Age | 0.14 | | 0.20 | | -0.18 | | 0.07 | | | -0.04 | 0.61 | -0.001 | | 0.68 | | | -0.18 | | 0.035 | | -0.01 | | 0.72 | | 0.004 | 0.56 |
|  | Sex | 7.79 | | 0.016 | | 10.26 | | 0.001 | | | 2.01 | 0.30 | -0.03 | | 0.74 | | | -1.59 | | 0.46 | | 5.12 | | <0.001 | | 1.14 | <0.001 |
|  | BMI | -0.28 | | 0.37 | | 0.22 | | 0.37 | | | -0.60 | 0.018 | -0.007 | | 0.56 | | | 0.69 | | 0.005 | | 0.26 | | 0.002 | | 0.12 | <0.001 |
|  | RPE | 1.09 | | 0.18 | | -2.30 | | 0.003 | | | 0.96 | 0.08 | -0.03 | | 0.25 | | | 1.21 | | 0.044 | | 0.009 | | 0.97 | | 0.02 | 0.64 |
|  |  |  |  | |  | |  | |  | | | | | | |  | | |  | |  | |  | |  | | |

As random effects the participant and residuals, as cluster effect the institution. For these parameters the variances are shown. The fixed effects are number of training sessions, mean motivation averaged over all training sessions, measurement day (PRE/POST), age of the participant, sex, body mass index (BMI) as well as RPE averaged over all training sessions.
